# The role of ROC75 as a daytime component of the circadian oscillator in *Chlamydomonas reinhardtii*

**DOI:** 10.1371/journal.pgen.1008814

**Published:** 2020-06-17

**Authors:** Takuya Matsuo, Takahiro Iida, Ayumi Ohmura, Malavika Gururaj, Daisaku Kato, Risa Mutoh, Kunio Ihara, Masahiro Ishiura

**Affiliations:** 1 Center for Gene Research, Nagoya University, Furo-cho, Chikusa-ku, Nagoya, Japan; 2 Graduate School of Science, Nagoya University, Furo-cho, Chikusa-ku, Nagoya, Japan; Ohio State University, UNITED STATES

## Abstract

The circadian clocks in chlorophyte algae have been studied in two model organisms, *Chlamydomonas reinhardtii* and *Ostreococcus tauri*. These studies revealed that the chlorophyte clocks include some genes that are homologous to those of the angiosperm circadian clock. However, the genetic network architectures of the chlorophyte clocks are largely unknown, especially in *C*. *reinhardtii*. In this study, using *C*. *reinhardtii* as a model, we characterized *RHYTHM OF CHLOROPLAST* (*ROC*) *75*, a clock gene encoding a putative GARP DNA-binding transcription factor similar to the clock proteins LUX ARRHYTHMO (LUX, also called PHYTOCLOCK 1 [PCL1]) and BROTHER OF LUX ARRHYTHMO (BOA, also called NOX) of the angiosperm *Arabidopsis thaliana*. We observed that ROC75 is a day/subjective day-phase-expressed nuclear-localized protein that associates with some night-phased clock genes and represses their expression. This repression may be essential for the gating of reaccumulation of the other clock-related GARP protein, ROC15, after its light-dependent degradation. The restoration of ROC75 function in an arrhythmic *roc75* mutant under constant darkness leads to the resumption of circadian oscillation from the subjective dawn, suggesting that the ROC75 restoration acts as a morning cue for the *C*. *reinhardtii* clock. Our study reveals a part of the genetic network of *C*. *reinhardtii* clock that could be considerably different from that of *A*. *thaliana*.

## Author summary

Circadian clock genes are not well-conserved during evolution, and the evolutionary history of circadian clocks is largely unknown. Green plants consist of two main clades: the Streptophyta (includes land plants and a part of freshwater green algae), and the Chlorophyta (includes a wide range of marine and freshwater green algae). Although the circadian clocks in chlorophyte algae include some components that are homologous to those of the circadian clock in flowering plants, it is not fully understood whether the chlorophyte clocks tick in the same manner as the flowering plant clocks. In this study, we characterized ROC75, a clock-related putative transcription factor, in the freshwater green alga *Chlamydomonas reinhardtii*. We found that ROC75 is a daytime repressor for the circadian clock in this alga, in contrast to its homologous protein, LUX (PCL1), which is known as a nighttime repressor for the flowering plant clock. Our study can serve as a step toward understanding the evolutionary history of circadian clocks in green plants.

## Introduction

Circadian clocks are self-sustained oscillators that confer adaptive advantages to organisms [1,2]. A transcriptional/translational autoregulatory feedback loop mechanism consisting of some specific genes, called the “clock genes,” plays a crucial role in the central oscillatory mechanism of these clocks [3]. Although the basic properties of circadian clocks are common among all model organisms that have been studied, the primary sequences of clock genes/proteins are not conserved across the kingdoms of life [3]. In addition, even within the organisms that have the same set of clock genes, the roles of some clock genes differ from those of others (for example, the roles of cryptochromes in mammalian and insect clocks are different) [3]. Although the clock genes and their genetic network architectures are quite variable, the evolutionary aspects of circadian clocks remain poorly understood.

Several studies, mainly on *Arabidopsis thaliana*, have revealed a complicated network architecture of the feedback loop mechanisms of clock genes in angiosperms [4,5]. At the core of the network, is a mutual genetic interaction between two dawn-phased MYB transcription factor genes, *CIRCADIAN CLOCK ASSOCIATED 1* (*CCA1*)/*LATE ELONGATED HYPOCOTYL* (*LHY*) and an evening-phased pseudo response regulator (*PRR*) gene called *TIMING OF CAB EXPRESSION 1* (*TOC1*) [6–9]. In addition, another evening-phased gene, *LUX* (also called *PCL1*), encoding a GARP DNA-binding transcription factor interacts with the core loop [10,11]. The LUX (PCL1) protein acts as a night-time repressor by forming a protein complex with EARLY FLOWERING (ELF) 3 and ELF4 [12,13], and represses *PRR9* whose product, in turn, represses *CCA1*/*LHY* [12,14]. LUX (PCL1) is thus an indirect activator of *CCA1*/*LHY*. BROTHER OF LUX ARRHYTHMO (BOA, also called NOX) is the other evening-phased clock-related GARP transcription factor that also forms a complex with ELF3 and ELF4 and promotes *CCA1* expression [13,15].

Green plants consist of two main clades: the Streptophyta (include the angiosperm *A*. *thaliana*), and the Chlorophyta [16]. In recent years, clock genes have been identified in two chlorophyte species, *Chlamydomonas reinhardtii* (Chlorophyceae) and *Ostreococcus tauri* (Prasinophyceae), and it was revealed that their circadian clocks include genes that are homologous to those of the *A*. *thaliana* clock [17–21]. In *O*. *tauri*, *CCA1* and *TOC1* homologs form a transcriptional circuit which can generate a robust circadian oscillation [22–24]. Therefore, the core transcriptional circuit of the *O*. *tauri* clock is likely to be a simplified version of that of the *A*. *thaliana* clock [20,21]. On the other hand, in *C*. *reinhardtii*, homologous genes have been identified using a forward genetic screen [25]. *ROC15* and *ROC75* (*ROC* stands for “*r**hythm* *o**f* *c**hloroplast* bioluminescence,” used as an indicator for the forward genetic screen) encode LUX (PCL1)/BOA (NOX)-like GARP proteins. *ROC40* encodes a CCA1/LHY-like MYB protein. However, their expression phases or phase relationships do not correspond exactly to those of the *A*. *thaliana* homologs [18,19,25]. Moreover, *ROC66*, encoding a CONSTANS-like (COL) protein, has a considerable impact on the *C*. *reinhardtii* clock [25] whereas only a limited effect has been reported by the misexpression of a COL family gene in the *A*. *thaliana* clock [26]. Collectively, it seems to be difficult to apply the *A*. *thaliana* model directly to the *C*. *reinhardtii* clock [18,19].

In addition, a considerable number of genes, which are not conserved in the *A*. *thaliana* genome or are conserved but have not been described as a clock component, are known to be involved in the circadian clock of *O*. *tauri* and *C*. *reinhardtii*. These genes include *LOV-HK* and *CPF1* of *O*. *tauri* [27,28], as well as *ROC55*, *ROC59*, *ROC114*, *NAT3*, *XRN1* (*ROC86*), *C1*, *C3*, and *pCRY* of *C*. *reinhardtii* [25,29–32]. It is noteworthy that cryptochrome genes (*CPF1* and *pCRY*) have a critical role in the circadian oscillators of these alga [27,32]. Cryptochromes are the core components of the mammalian circadian oscillator [3]. While mammalian cryptochromes and CPF1 belong to a phylogenetic clade called “animal-CRY,” pCRY belongs to “plant-CRY” with the *A*. *thaliana* cryptochromes, CRY1 and CRY2, which are involved in the input of light information to the circadian oscillator but not in the oscillator itself [33,34]. Taken together, it is likely that the chlorophyte algae have developed their own mechanisms different from angiosperms to adapt to their environment during evolution.

Here, we investigated the network architecture of the circadian transcriptional loop of *C*. *reinhardtii* to shed some light on the evolution of circadian clocks in green plants. We focused on the GARP protein gene, *ROC75*, in *C*. *reinhardtii*. *ROC75* shows daytime mRNA expression [25], whereas *ROC15*, the other clock-related GARP protein gene in *C*. *reinhardtii*, shows nighttime mRNA (and protein) expression [35] similar to *LUX* (*PCL1*)/*BOA* (*NOX*) in *A*.*thaliana* [10,11].

## Results

### *roc75* is a conditional mutant

**Fig 1:**
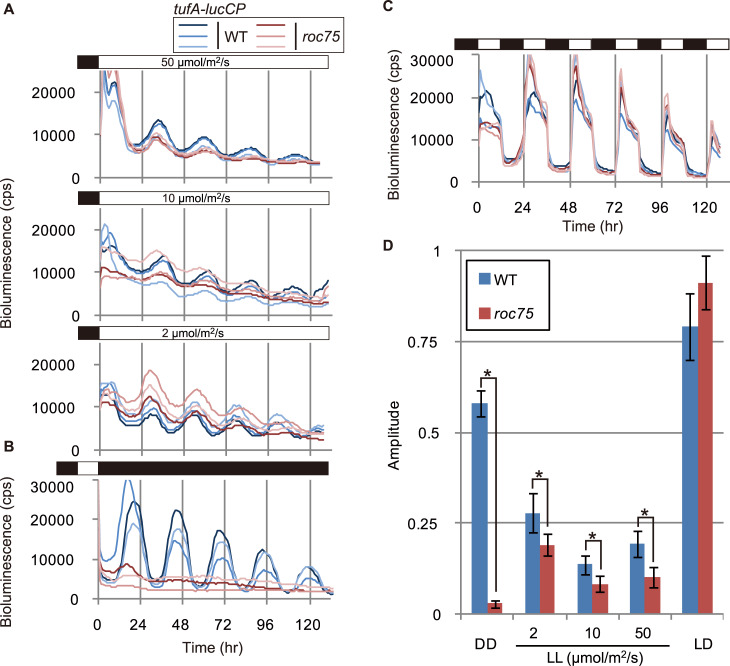
Bioluminescence rhythms of the *roc75* mutant. **(A**, **B**, **C)** Bioluminescence rhythm assay for the *tufA-lucCP* reporter gene introduced into the chloroplast genome under LL, DD, and LD conditions. Three representative bioluminescence traces of the WT and *roc75* cells are shown. White and black bars on the top of graphs represent light and dark conditions, respectively. **(D)** Amplitude of the rhythm. Bars represent means ± standard deviation (SD) of 6–18 biologically independent measurements. The asterisks indicate statistically significant differences (Student *t*-test; *P* < 0.001).

Previously, we had isolated *roc75*, which shows arrhythmicity under constant darkness (DD) [25] in the bioluminescence rhythm derived from the *tufA-lucCP* reporter gene introduced into the chloroplast genome [36]. Here, we tested the bioluminescence reporter rhythm under continuous light (LL) conditions using various light intensities (50, 10, and 2 μmol/m^2^/s) and in a light/dark (LD; 12-h light:12-h dark) cycle. Under the LL conditions, *roc75* showed bioluminescence rhythms with lower amplitudes than those of the wild-type (WT) strain at all the light intensities tested (Fig 1A and 1D), indicating that *ROC75* is required for the normal rhythmicity of the reporter under LL conditions, as well. However, *roc75* showed rhythmicity under the LL conditions unlike the arrhythmicity it showed under the DD conditions (Fig 1B and 1D). In addition, a diurnal bioluminescence rhythm, which appeared normal, was detected in *roc75* under the LD condition (Fig 1C and 1D). This rhythm cannot be explained by a simple light/dark response of the reporter, since another arrhythmic mutant *roc114* [25] showed a lower amplitude of the bioluminescence rhythm under the same condition (S1A and S1B Fig). Taken together, these results indicate that *ROC75* is indispensable for circadian rhythmicity under DD conditions but not in the presence of light. This implies that the function of *ROC75* is compensated by light to some extent.

### ROC75 is a day/subjective day-phase-expressed nuclear-localized protein

**Fig 2:**
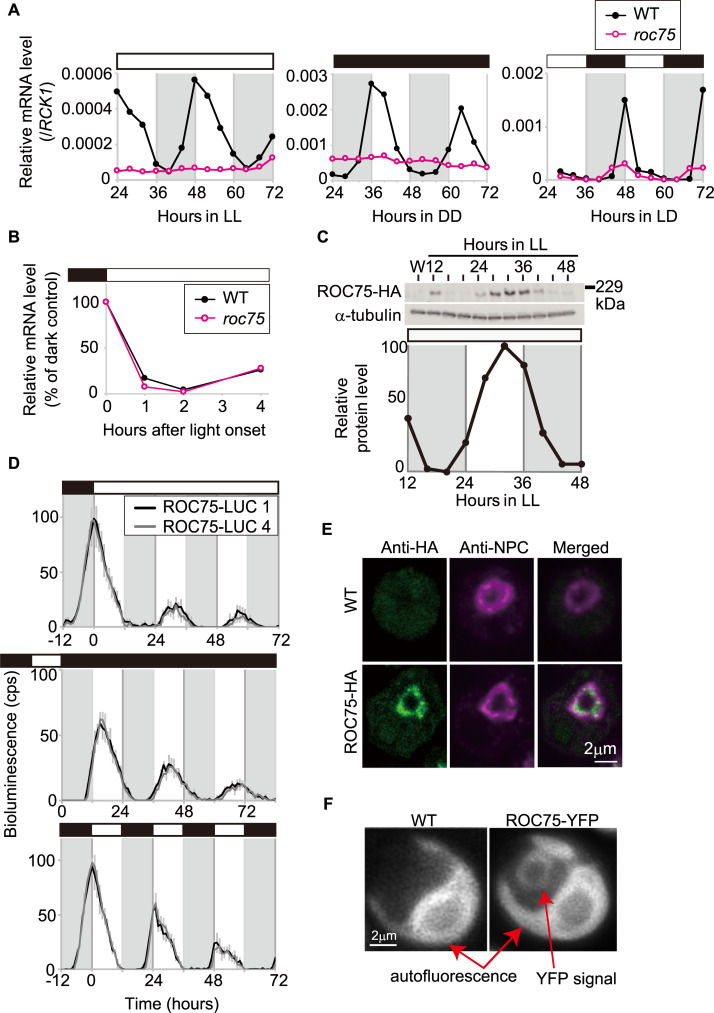
Expression analyses of *ROC75* gene. **(A)** RT-qPCR analysis of the *ROC75* mRNA rhythm under LL, DD, LD conditions. The cells from the batch HS cultures were used. **(B)** RT-qPCR analysis of the *ROC75* mRNA in response to light. The cells from the LD-entrained batch HS cultures were harvested at 1, 2, and 4 h after switching-on the lights. The relative values with respect to the dark control cells at each time point are shown. **(C)** Western blot analysis of the ROC75-HA protein. Total protein samples were extracted from a continuous culture of ROC75-HA strain exposed to LL conditions. Western blot of α-tubulin is shown as a loading control. The lysate of the WT strain (W) was used as a negative control. **(D)** Bioluminescence rhythm assay of the ROC75-LUC protein fusion reporter. The traces represent means ± SD of more than five biological replicates. **(E)** Immunocytochemical staining of ROC75-HA. The cells were harvested from batch TAP cultures of the ROC75-HA strain at L4 (4 h after switching-on the lights). Counterstaining with the NPC antibody and merged images are indicated. **(F)** YFP fluorescence of ROC75-YFP. Batch TAP cultures of the ROC75-YFP strain were transferred to DD conditions, and YFP fluorescence was observed at 18 h after the transfer (subjective midday). White and black bars above the graphs represent the light and dark conditions, respectively. The shaded areas in the main body of the graphs represent the dark period under the LD condition or the time of day corresponding to the dark period for synchronization before the exposure to LL and DD conditions. Representative results are shown (A-F). The reproducibility was confirmed by performing at least two independent experiments (A-F) and by using more than two independent transgenic lines (C-F).

We analyzed the temporal and spatial expression profiles of ROC75. As reported in a previous study [25], the expression of *ROC75* mRNA was high during the early subjective day under LL conditions (Fig 2A). A similar circadian profile was observed under DD conditions (Fig 2A). Under the LD cycle, the *ROC75* mRNA showed a sharp peak at dawn (Fig 2A). The acute decline after the peak was due to a downregulation by light (Fig 2B). These mRNA rhythms were blunted for the mutated *ROC75* mRNA (having a large insertion of the *aph7"* gene [25]) in *roc75* (Fig 2A).

To analyze the expression of the ROC75 protein, we introduced a codon-adapted hemagglutinin (HA) epitope tag sequence [35] at the end of the coding sequence of a genomic DNA fragment of *ROC75* locus, and *roc75* mutant was transformed with the DNA fragment (S2A Fig). The tagged gene fully complemented the arrhythmicity of *roc75* under DD conditions, indicating that the HA-tagged ROC75 was functional (S2B Fig). The ROC75-HA protein showed a rhythm, peaking at the subjective day under LL conditions (Fig 2C). Furthermore, bioluminescence assay by using a luciferase reporter translationally fused to the C-terminal of full length ROC75 protein (ROC75-LUC) [35] and the luciferin analog AkaLumine (see Materials and Methods) also indicated diurnal/circadian rhythms peaking at the day/subjective day under LL, DD, and LD conditions (Fig 2D). These results suggest that ROC75 is a day/subjective day-phase-expressed protein. In addition, because ROC75-LUC complemented the arrhythmicity of *roc75*, the ROC75-LUC fusion protein is probably functional as a clock component (S3 Fig).

To determine the intracellular localization of ROC75, we performed immunocytochemical staining of ROC75-HA. The ROC75-HA signal was detected just inside of the nuclear envelope, which was counterstained with an antibody for the nuclear pore complex (NPC) (Fig 2E). In addition, we detected similar nuclear signals from the ROC75-YFP fusion protein expressed using the same strategy that was employed for ROC75-HA (Fig 2F, S2 Fig). Taken together, these results suggest that ROC75 is a day/subjective day-phase-expressed nuclear-localized protein.

### ROC75 associates with the rhythmic circadian clock genes

**Fig 3:**
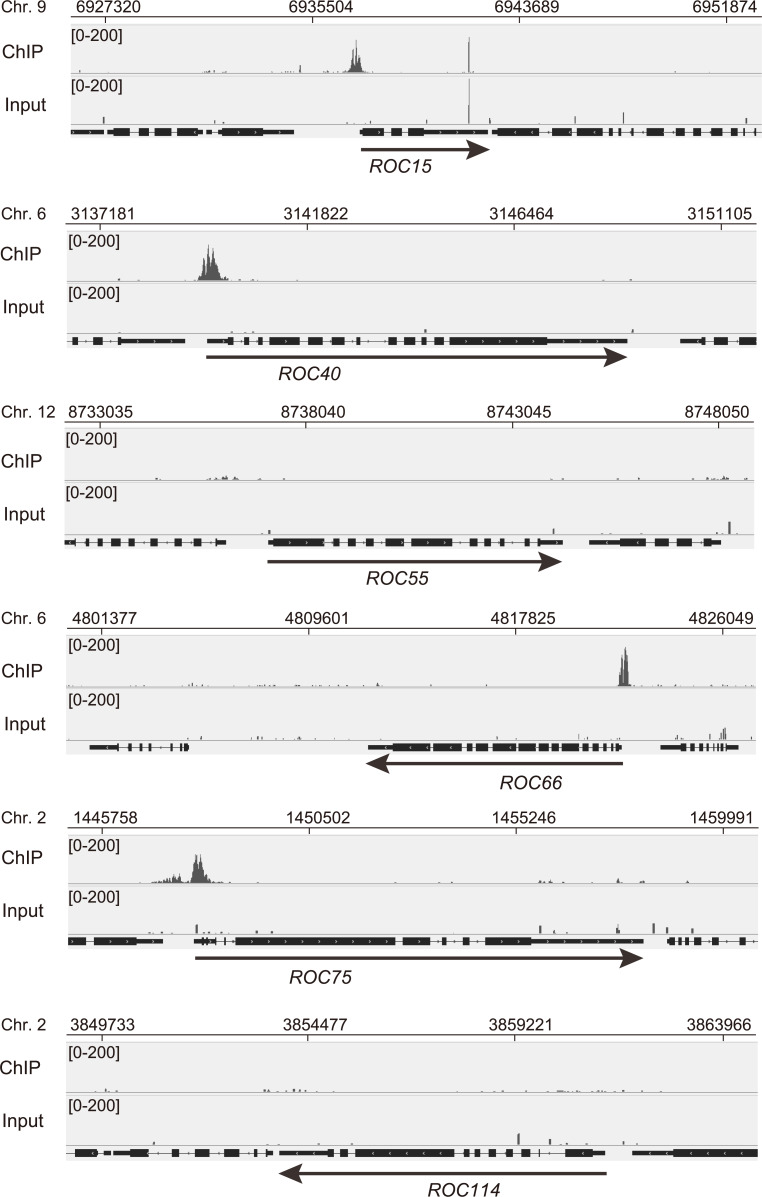
ChIP-Seq analysis of ROC75-HA. The ROC75-HA cells were harvested from batch TAP cultures at L4 (4 h after switching-on the lights). Data shown are histograms of the number of mapped sequence tags around the clock genes. The results for the input DNA are shown as negative control.

Because ROC75 has a GARP DNA-binding motif [25], we investigated its association with DNA using chromatin immunoprecipitation followed by deep sequencing (ChIP-seq). We could detect 81 peaks of ROC75-HA associated DNA fragments on the nuclear genome (S1 Table). Most of the peaks were located in the upstream region (36/81) and in the 5′-untranslated region (UTR) (31/81) of the target genes (S1 Table, S4A Fig). A consensus motif search for the sequences around the 81 peaks identified “GATTYKV” (S4B Fig), which is a close match to the consensus motif of *A*. *thaliana* LUX (PCL1) (GATWCC, [12]). The peaks were detected on the clock genes (*ROC15*, *ROC40*, *ROC66*, and *ROC75* itself) (Fig 3) that are known to show circadian rhythms in their mRNA levels [25]. On the other hand, peaks were not detected on non-rhythmic *ROC* genes (*ROC55* and *ROC114* [25]) (Fig 3). The ChIP-seq peak on the *ROC40* gene was confirmed by ChIP followed by quantitative PCR (ChIP-qPCR) (S5 Fig). A peak was also detected on *ROC59* (S1 Table, Peak No. 5), which is another *ROC* gene that was identified as the gene responsible for a mutant that showed “long-period” and “delayed-phase” circadian phenotypes [25]. In addition, a peak was detected on *CrPRR1*, a PRR-like gene encoding a protein similar to the *A*. *thaliana* TOC1 [37], but has not yet been described as a clock component in *C*. *reinhardtii* (S1 Table, Peak No. 23).

The number of peaks detected was relatively low, probably because of technical reasons. We consider that our ChIP-seq assay covered only highly specific- but not the entire-fraction of ROC75 targets. Therefore, in this study, we focused only on the clock and putative clock genes that were detected.

We tested the DNA-binding activity of the GARP motif of ROC75 by electrophoresis mobility shift assay (EMSA). We used a GST-tagged GARP DNA-binding domain protein of ROC75 (GST-GARP) purified from *Escherichia coli* (S6A Fig) and a 26-bp DNA fragment from the amplicon 3 of *ROC40* (S5 Fig) containing the ChIP-seq consensus sequence, GATTYKV (S4B Fig). A mobility shift was observed in the WT DNA fragment (S6B Fig, aGATTTTCt). This binding was weakened by a mutation in the first GAT (S6B Fig, a**CCC**TTTTCt) but not by a mutation in its neighboring nucleotides (S6B Fig, **c**GAT**C**TTCt), and was abrogated by a mutation in both of them (S6B Fig, **c****CCCC**TTCt). This result indicates that the GARP domain of ROC75 can bind to DNA directly in a sequence-specific manner.

### ROC75 represses the clock-related genes

**Fig 4:**
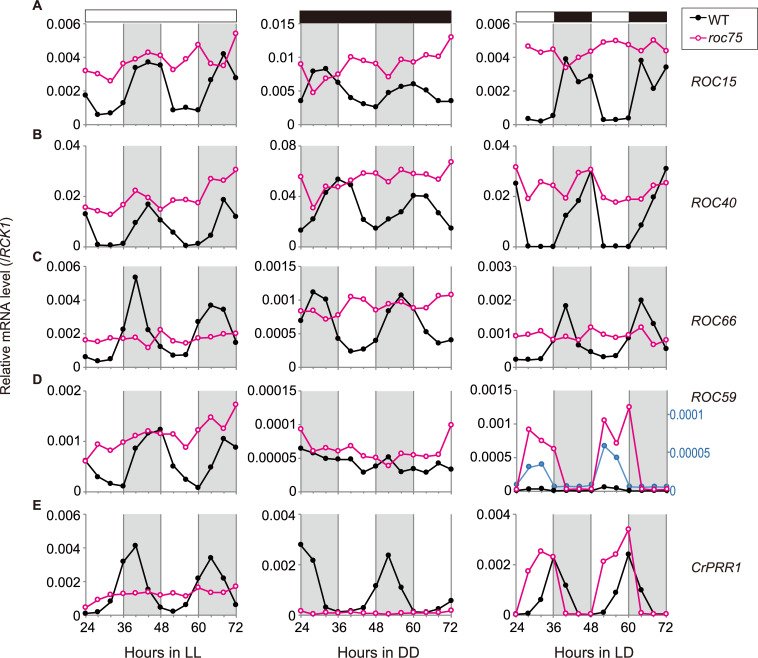
The mRNA rhythm of clock-related genes. **(A-E)** RT-qPCR analysis of the mRNA rhythm of the clock-related genes. The cells were harvested from batch HS cultures exposed to LL, DD, and LD conditions. White and black bars above the graphs represent the light and dark conditions, respectively. The shaded areas in the main body of the graphs represent the dark period under the LD condition or the time of day corresponding to the dark period for synchronization before the exposure to LL and DD conditions. The magnified trace of *ROC59* expression in WT under the LD condition is shown in blue, and its y-axis is shown on the right (D). Representative results are shown. The reproducibility was confirmed by performing at least two independent experiments.

We tested the effect of *roc75* mutation on the expression of the target genes identified by ChIP-seq analysis at the mRNA level. As described previously [25], the mRNA levels of *ROC15*, *ROC40*, and *ROC66* showed robust circadian rhythms, peaking at the subjective night under LL conditions in the WT strain (Fig 4A–4C, LL). Essentially the same rhythmic expression patterns were observed under DD and LD conditions (Fig 4A–4C, DD and LD). In the *roc75* mutant, the downregulation of the expression of these genes during the day/subjective day was weak or was almost abolished under all the conditions tested (Fig 4A–4C). These results suggest that ROC75 acts as a repressor of these genes during the day/subjective day. In the case of *ROC66*, however, the mRNA level was notably low in the mutant during the night/subjective night, especially under LL and LD conditions (Fig 4C), suggesting the lack of an activation mechanism or suggesting the involvement of additional repressor(s) other than ROC75.

The expression of *ROC59* was highly dependent on the light conditions. The expression under DD conditions was one order of magnitude lower than that under the LL conditions, and no rhythmicity was detected both in the WT strain and the *roc75* mutant (Fig 4D, DD). Under the LD condition, a peak was observed in the day, and the peak expression level was high in the *roc75* mutant (Fig 4D, LD), suggesting that ROC75 acts as a repressor of the light-dependent expression of *ROC59* during the day. Under LL conditions, the expression level was high in both the WT strain and *roc75* mutant, and the downregulation during subjective day was abolished in the *roc75* mutant (Fig 4D, LL). These results suggest that ROC75 acts as a repressor of the light-dependent expression of *ROC59*.

The expression of *CrPRR1* in the WT strain showed a circadian rhythm, peaking in the evening, under the LD condition and in the early subjective night under LL and DD conditions (Fig 4E). In the *roc75* mutant, the expression during the day/subjective day under LD and LL conditions was higher than in the WT strain (Fig 4E, LD and LL), again suggesting a repressor function of ROC75 during the day/subjective day. Also, the lower expression level in *roc75* during the subjective night under LL conditions suggests the involvement of other regulation mechanisms as in the case of *ROC66*. Interestingly, however, the expression of *CrPRR1* was very weak in the *roc75* mutant under DD conditions and during the dark phase under the LD condition (Fig 4E, DD and LD). This indicates that ROC75 is required for the activation of *CrPRR1* expression in the dark.

### Acute downregulation of *ROC15* and *ROC40* mRNAs by light is independent of ROC75

**Fig 5:**
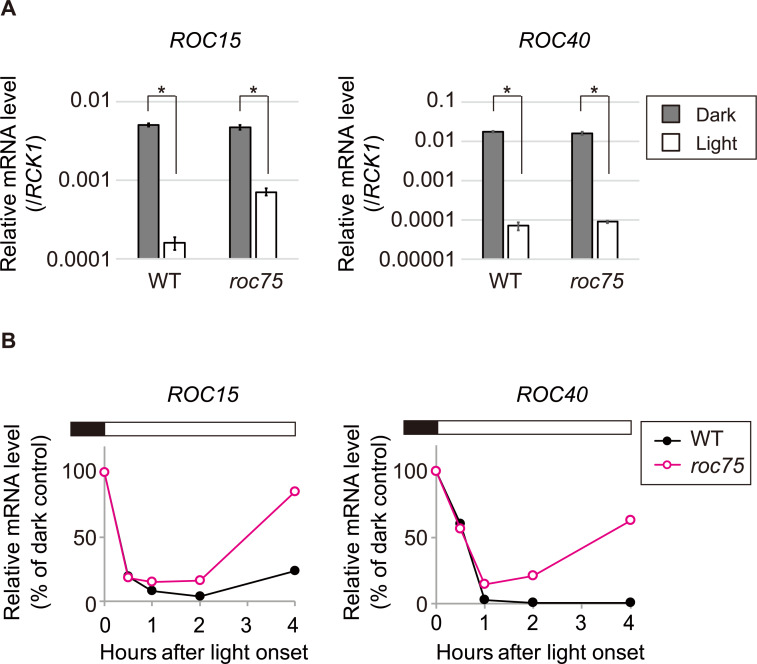
Light response of *ROC15* and *ROC40* mRNAs. **(A)** RT-qPCR analysis of the acute light response. LD-synchronized batch HS cultures were exposed to light (30 μmol/m^2^/s) at the late night (ZT22, 2 h before dawn). One hour after the switching-on of lights, the cells were harvested along with the control cells that were not exposed to light. Data indicate means ± SD of three biological replicates. The asterisks indicate statistically significant differences (Student *t*-test; *P* < 0.001). **(B)** RT-qPCR analysis of the light response at dawn. LD-synchronized batch HS cultures were harvested at 0 (dark), 1, 2, and 4 h after light onset. Data shown are the relative values with respect to dark controls that were kept under dark conditions. White and black bars above the graphs represent the light and dark conditions, respectively. Representative results are shown. The reproducibility was confirmed by performing at least two independent experiments.

In addition to endogenous oscillations, the *ROC15* and *ROC40* mRNAs show an acute decline after exposure to light [35,38,39]. To investigate this light response in *roc75* to light, cells in the late night phase were exposed to light for 1 h. An acute decline in the expression of these mRNAs was observed in the *roc75* mutant as well as in the WT strain (Fig 5A). The acute decline also occurred after the dawn under the LD condition but the levels of these mRNAs were recovered in the *roc75* mutant (Fig 5B). These results indicate that ROC75 is not essential for the acute downregulation of *ROC15* and *ROC40* mRNAs by light, but has a role in preventing their reaccumulation during the day.

### Rhythms of clock proteins are abolished by *roc75* mutation under DD but not under LL and LD conditions

**Fig 6:**
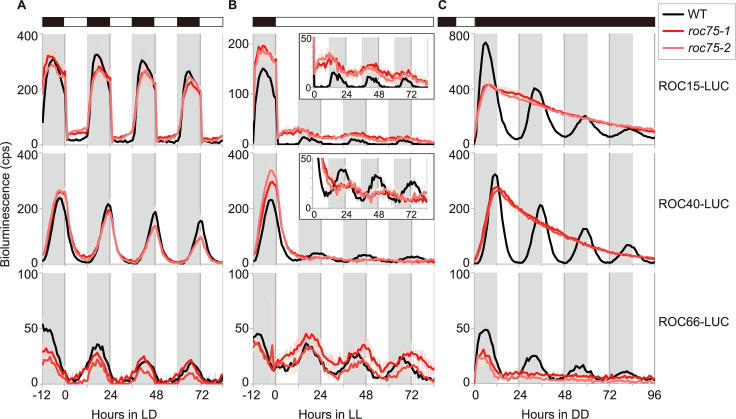
Rhythms of clock-related proteins. **(A-C)** Bioluminescence rhythm assay of the ROC15-LUC, ROC40-LUC, and ROC66-LUC protein fusion reporters under LD (A), LL (B), and DD (C) conditions. The reporters having *roc75* mutation were obtained by genetic crosses between the mating type (mt)^+^ reporter strains and mt^-^ strain of the *roc75* mutant. The result from two mutant progenies and parental WT reporter strains are shown. The traces represent the means ± SD of at least five biological replicates. The insets are magnified graphs. White and black bars above the graphs represent the light and dark conditions, respectively. The shaded areas in the main body of the graphs represent the dark period under the LD condition or the time of day corresponding to the dark period for synchronization before the exposure to LL and DD conditions.

To assess the effect of the *roc75* mutation on the rhythms of clock proteins, we measured the bioluminescence rhythms of luciferase reporters translationally fused to ROC15, ROC40, and ROC66 proteins (ROC15-LUC, ROC40-LUC, and ROC66-LUC respectively [35]). Under the LD condition, in sharp contrast to the mRNA data, the bioluminescence rhythms of these reporters were similar to that of WT in the *roc75* mutant (Fig 6A). Under LL conditions, although the rhythm patterns were disturbed in the *roc75* mutant, these reporter signals were still rhythmic (Fig 6B). Under DD conditions, however, these reporter signals were completely arrhythmic in the *roc75* mutant, and the bioluminescence levels of ROC15-LUC and ROC40-LUC were constantly high (Fig 6C). These results suggest that the deregulation of the rhythms of clock mRNAs in the *roc75* mutant were reflected in the rhythms of their respective proteins under DD but not under LL and LD conditions. This result is in good agreement with the conditional arrhythmicity of the bioluminescence rhythm in chloroplast reporter strain (Fig 1).

Essentially same results were obtained in an *in vitro* luciferase assay using crude extracts of the reporter cells under DD conditions (S7 Fig), ruling out the possibilities that the results of the *in vivo* bioluminescence assay were because of the changes in the availability of intracellular ATP, luciferin, magnesium ion, and/or oxygen in this mutant.

### Loss of circadian gating of ROC15 reaccumulation in the *roc75* mutant

**Fig 7:**
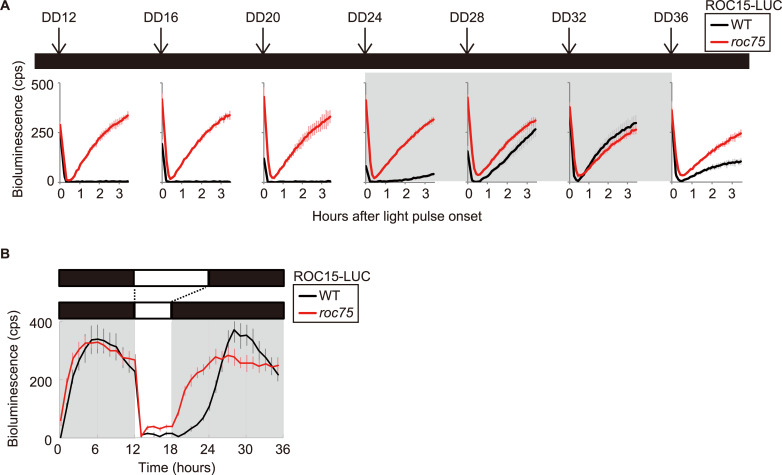
Light response of the ROC15 protein. **(A)** Bioluminescence assay of ROC15-LUC for recovery after exposure to a light pulse. The ROC15-LUC reporter was exposed to a light pulse (5 min, 10 μmol/m^2^/s) at the time-points indicated under DD conditions. **(B)** Bioluminescence assay of ROC15-LUC for response to a premature darkness. The LD-synchronized ROC15-LUC reporter strains were exposed to 6-h premature darkness. The traces represent means ± SD of at least five biological replicates (A, B). White and black bars above the graphs represent the light and dark conditions, respectively. The shaded areas in the main body of the graphs represent the time of day corresponding to the dark period for synchronization before the exposure to DD conditions (A) and the dark period (B).

It is obvious that the light-induced acute degradation of ROC15 at dawn occurred normally in the *roc75* mutant (Fig 6A, ROC15-LUC), indicating that ROC75 is not involved in this process. Previously, we demonstrated that the recovery of the ROC15 level after degradation was restricted to the subjective night [35]. Interestingly, the recovery was observed throughout the circadian cycle in the *roc75* mutant (Fig 7A). In addition, the *roc75* mutant failed to keep low ROC15-LUC bioluminescence level during the subjective day when the cells were exposed to a premature darkness (Fig 7B). These results suggest that ROC75 contributes to the circadian gating of ROC15 reaccumulation.

### Repression- but not activation-domain fusion is compatible with ROC75

**Fig 8:**
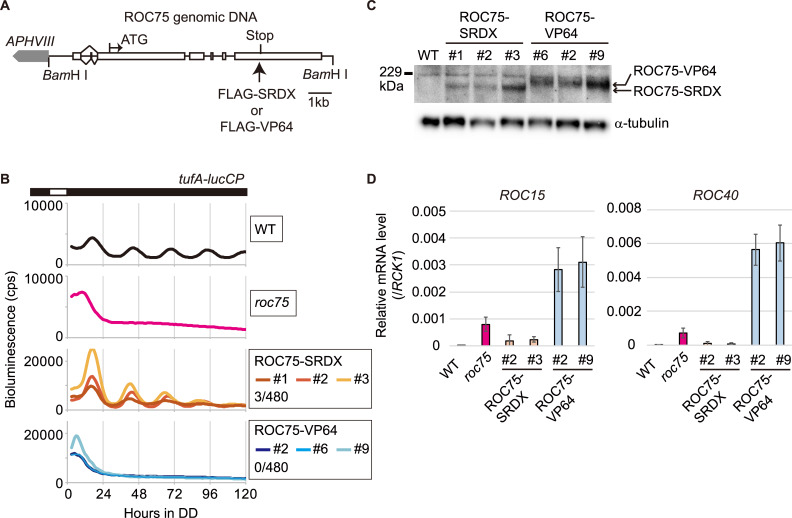
Fusion of repression and activation domains to ROC75. **(A)** A schematic representation of the ROC75-SRDX and ROC75-VP64 transgenes. White boxes represent exons. The positions of the 5′- and 3′-ends of the transcript are based on the latest genome annotation (*Chlamydomonas reinhardtii* v5.6, Phytozome 13, https://phytozome-next.jgi.doe.gov/). **(B)** Bioluminescence rhythm assay of the *tufA-lucCP* reporter in ROC75-SRDX and ROC75-VP64 strains. Each trace represents the average bioluminescence level of two biological replicates. The number in the graph legend represents complemented-transformant/total-transformant-tested. **(C)** Western blot analysis of the ROC75-SRDX and ROC75-VP64 proteins. The cells were harvested from batch TAP cultures at dawn, α-tubulin is shown as a loading control. Anti-FLAG antibody was used to detect these proteins. **(D)** RT-qPCR analysis of *ROC15* and *ROC40*. The cells were harvested from batch TAP cultures at L4 (4 h after switching-on the lights). Data indicate means ± SD of three biological replicates.

To investigate the molecular function of ROC75, we tested the compatibility of ROC75 with repression- and activation-domain fusion. Fusion to a domain, having the same transcriptional function (repression or activation) as ROC75, is expected to enhance or not interfere with its function. Conversely, fusion to a domain having an opposite function is expected to interfere with it. We employed the strong repression domain SRDX [40] and the strong activation domain VP64 [41]. Codon-adapted sequences of the domains for the *C*. *reinhardtii* nuclear genome (S8 Fig) were inserted at the end of the coding sequence of the ROC75 genomic DNA fragment to express the C-terminally-tagged version of ROC75 (ROC75-SRDX and ROC75-VP64) (Fig 8A). ROC75-SRDX complemented the arrhythmicity of *roc75* mutant at a rate comparable to that of ROC75-HA and ROC75-YFP (Fig 8B, S2 Fig). On the contrary, no transformant with ROC75-VP64 showed restoration of circadian rhythmicity even in transformants expressing the fusion protein at a similar level to ROC75-SRDX (Fig 8B and 8C). Furthermore, upregulation of *ROC15* and *ROC40* mRNAs was observed in ROC75-VP64 transformants in the day phase (Fig 8D), suggesting that the VP64 domain fusion is functional in *C*. *reinhardtii*. These results suggest that ROC75 acts as a transcriptional repressor.

### Restoration of ROC75 function resumes the circadian oscillation from subjective dawn

We examined the effect of timing of restoration of ROC75 function in the arrhythmic *roc75* mutant in DD. For this purpose, we took advantage of the glucocorticoid receptor (GR) system to control the activity of ROC75. The GR system has been developed to regulate the activity of transcription factors in *A*. *thaliana*. Transcription factors fused to the 287-aminio-acid hormone binding domain of GR cannot regulate transcription in the absence of the steroid ligand because of association with a heat-shock protein 90 complex, but the transcriptional activity is restored by adding the ligand dexamethasone (DEX), which induces a conformational change in GR, subsequently releasing the fusion protein from the complex [42].

**Fig 9:**
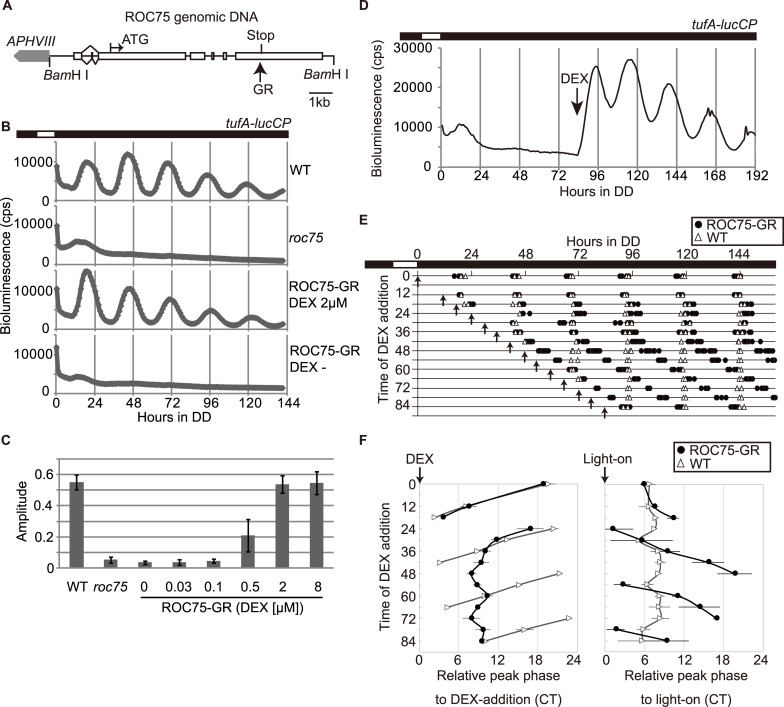
Restoration of ROC75 function by the GR-system. **(A)** A schematic view of the ROC75-GR transgene. White boxes represent exons. The positions of the 5′- and 3′-ends of the transcript are based on the latest genome annotation (*Chlamydomonas reinhardtii* v5.6, Phytozome 13, https://phytozome-next.jgi.doe.gov/). **(B)** Bioluminescence rhythm assay of the *tufA-lucCP* reporter in ROC75-GR strain. DEX was added to a final concentration of 2 μM just before synchronization. The DEX- control was treated with the same concentration of the solvent (EtOH). **(C)** Amplitude of the bioluminescence rhythm. Each bar represents the mean ± SD of six biological replicates. **(D)** A representative trace of the bioluminescence of the ROC75-GR strain before and after DEX addition. DEX (final concentration: 2 μM) was added at the 84-h time-point under DD conditions. **(E)** The phase of ROC75-GR bioluminescence rhythm after DEX addition. The peaks of each bioluminescence trace of ROC75-GR (duplicate cultures of four independent transgenic lines) and WT (2–4 cultures) were plotted on the graph. The arrows indicate the timing of DEX addition (final concentration: 2 μM). All representative traces are shown in S10 Fig. **(F)** Peak phase relative to the DEX-addition and light-on. The phase in circadian time (CT) was calculated against the timing of DEX-addition (left) and switching-on of the light in LD cycle preceding the exposure to DD condition (right). Data points represent means ± SD of four and six to eight biological replicates of the WT and ROC75-GR (from four independent transgenic lines), respectively. White and black bars above the graphs represent light and dark conditions, respectively (B, D, E).

A codon-adapted sequence of the hormone binding domain of rat GR for the *C*. *reinhardtii* nuclear genome (S9 Fig) was inserted at the end of the coding sequence of the ROC75 genomic DNA fragment to express the C-terminally tagged version of ROC75 (ROC75-GR) (Fig 9A). ROC75-GR complemented the *roc75* mutant phenotype in the presence of DEX but not in the absence of DEX (Fig 9B). The restoration of the amplitude of bioluminescence rhythm was dependent on the dose of DEX (Fig 9C). These results indicate that the GR system can regulate the activity of ROC75 in *C*. *reinhardtii*.

By adding DEX to arrhythmic ROC75-GR cultures under DD conditions, the bioluminescence rhythms were restored (Fig 9D). We added DEX at various times under the DD conditions, and analyzed the circadian phase of the recovered bioluminescence rhythm (Fig 9E, S10 Fig). The results were different between the first day and the second or later days under DD conditions. When DEX was added on the first day, the restored rhythms showed phases relatively close to those of the WT rhythms (Fig 9E and 9F). In other words, the phase was mostly dependent upon the LD cycle before transfer to DD conditions, indicating that the previous light/dark cue is dominant to the ROC75 restoration during this period. In contrast, when DEX was added on the second or later days, the phase of the restored rhythms was dependent upon the timing of DEX addition (Fig 9E and 9F). The peak phase relative to DEX addition was 8–10 h (Fig 9F, left), which was comparable to that relative to the light-on in the WT strain (Fig 9F, right), suggesting that DEX acts as a morning cue in this system.

## Discussion

### Roles of ROC75 in the *C*. *reinhardtii* circadian clock

In the *roc75* mutant under DD conditions, the expression of *ROC15* and *ROC40* was almost constantly high (Fig 4A and 4B, DD; Fig 6C), similar to their expression state just before the subjective dawn in the WT cells. This suggests that the circadian clock in the *roc75* mutant is arrested before the subjective dawn because of the loss of downregulation of these genes by ROC75. This hypothesis is consistent with the result of DEX acting as a morning cue (Fig 9E and 9F, after the second day under DD conditions). These results suggest that one of the roles of ROC75 is its involvement in the progression of the circadian clock from the subjective night to the subjective day under DD conditions. In addition, the loss of circadian gating of ROC15 reaccumulation (Fig 7) suggests that another role of ROC75 is the maintenance of the "subjective day state" of the circadian clock by preventing reaccumulation of the nighttime clock protein ROC15. This gating might be supported, at least in part, by the repression at the mRNA level by ROC75 (Fig 4A, DD).

In contrast to DD conditions, the circadian rhythms of clock proteins were robust in the LD condition in the *roc75* mutant (Fig 6A). It is possible that the acute downregulations in *ROC15* and *ROC40* mRNAs and ROC15 protein by light (Figs 5 and 7), which are independent of ROC75, generate the robust oscillation of the clock proteins, and compensate the ROC75 functions. Since the mRNA oscillations in the LD condition were weak or not detectable in the *roc75* mutant (Fig 4A and 4B, LD) probably due to their rapid recovery after transition to the day phase (Fig 5B), the contribution of the mRNA rhythms on the protein rhythms would be limited. Translational/posttranslational regulations, such as light-dependent degradation of ROC15, probably contribute more to the robust protein oscillations (Fig 6A). However, it is obvious that ROC75 has a role to keep *ROC15* and *ROC40* mRNA levels low during the day phase after their light-dependent downregulations at dawn (Figs 4A and 4B, 5B). This role is likely to contribute to prevent ROC15 protein accumulation in premature dark conditions (Fig 7B), suggesting that the second role of ROC75, i.e. maintenance of the subjective day state, is significant for the circadian clock even in the LD condition. In natural LD cycles, the light period is often disturbed by darkness due to cloudiness or shade. ROC75 may contribute to keeping time by preventing unexpected ROC15 accumulation in such situations.

### Possible additional components of the transcriptional loop of *C*. *reinhardtii* circadian clock

*ROC59* and *CrPRR1* are noteworthy among the target genes of ROC75. Although *ROC59* has already been identified as a clock-related gene [25], genetic interaction with *ROC75* strengthens the importance of *ROC59* in the *C*. *reinhardtii* clock. *ROC59* encodes a protein harboring several WD40 and tetratricopeptide repeats similar to the evolutionarily conserved antiobesity genes, *ASG2* (Altered Seed Germination 2) in higher plants, *ADP* (antiobesity factor ADIPOSE) in flies, and *WDTC1* (WD40 and TPR 1) in humans [25,43]. These genes are known to be involved in fat metabolism [43], but none of them has yet been described as a clock component. Because WDTC1 is known to regulate the transcriptional activities of the genes involved in adipogenesis through histone modifications [44,45], ROC59 might have a function in the epigenetic regulation of the circadian clock in *C*. *reinhardtii*.

The *CrPRR1* gene encodes a homolog of the *O*. *tauri* and *A*. *thaliana* clock protein, TOC1 [6,22]. The expression of *CrPRR1* peaked at dusk under LD condition and at early subjective night under LL and DD conditions (Fig 4E), indicating that the evening-phased expression is conserved in *C*. *reinhardtii*. Investigating whether *CrPRR1* is involved in the circadian oscillator function or one of the output pathways from the oscillator in the *C*. *reinhardtii* clock would be necessary in understanding the evolutionary history of circadian clocks in green plants.

### GARP transcription factors in the plant circadian clocks

In this study, we demonstrate that ROC75 is a daytime component of the *C*. *reinhardtii* clock that represses the mRNA expression of night-phased clock genes (e.g., *ROC15* and *ROC40*). This is in contrast to the case of *A*. *thaliana* LUX (PCL1) which is a nighttime repressor [12]. The features of ROC75 and LUX (PCL1) are summarized in Table 1. They also show sharp contrasts in their mutant phenotypes: An arrhythmic phenotype was observed in the *roc75* mutant under DD conditions (Fig 1B and 1D) [25] but in *lux/pcl1* mutants, it is observed under LL conditions [10,11]. In addition, a weak rhythm phenotype was observed under LL conditions in *roc75* (Fig 1A and 1D) but under DD conditions in the *CCR2*::*LUC* reporter in *lux* mutant [10]. On the other hand, both the mutants (*roc75* and *lux*) showed normal rhythm under LD conditions at least in some of the processes (Figs 1C, 1D and 6A) [10]. Meanwhile, *O*. *tauri* genome [46] has GARP protein genes, and one of them encodes a LUX-like protein (ostta12g01420). Although mutant phenotypes and the effect of misexpression have not been reported to date, the mRNA expression of this gene is evening-phased under LD conditions [47], similar to that of *LUX* (*PCL1*) and *BOA* (*NOX*) [10,11,15], in contrast to that of *ROC75* (Fig 2A, 2C and 2D) [25]. One possibility is that *C*. *reinhardtii* has developed a contrasting mechanism of operating the circadian clock to that of *A*. *thaliana* and *O*. *tauri*. Consistent with this notion, the effect of changes in the casein kinase 1 activity on the circadian clock also shows a sharp contrast; the circadian rhythm is shortened by an RNAi-mediated knockdown in *C*. *reinhardtii* [48], whereas it is lengthened by specific inhibitor treatments or misexpression in *A*. *thaliana* and *O*. *tauri* [49,50].

**Table 1:** Comparison of ROC75 and LUX (PCL1).

			ROC75	LUX (PCL1)	reference
**Protein expression**			Day	Night	13
Binding consensus*			GATTYKV	GATWCG	12
**Function**			Repressor	Repressor	12
**Mutant phenotypes**	Rhythm	DD	Arrhythmic	Weak	10
		LL	Weak	Arrhythmic	10, 11
		LD	Normal	Normal	10
	MYB gene expression**		Upregulated	Downregulated	10, 11

*: W = A or T; Y = C or T; K = G or T; V = A, C, or G

**: *ROC40* of *C*. *reinhardtii* and *CCA1*/*LHY* of *A*. *thaliana*

It is still unclear to what extent the *C*. *reinhardtii* clock is similar to the other plant clocks in the network architectures of the circadian transcriptional loops. This study indicates that at least the relationship between GARP proteins (ROC75 and LUX [PCL1]) and MYB transcription factor genes (*ROC40* and *CCA1/LHY*) is different between *C*. *reinhardtii* and *A*. *thaliana*. In *C*. *reinhardtii*, *ROC40* is a direct target of ROC75, and therefore, its expression is constantly high in the *roc75* mutant (Fig 4B). In contrast in *A*. *thaliana*, the expression of *CCA1/LHY* is indirectly regulated by LUX (PCL1) via PRR9 [12]. Since PRR9 is a transcriptional repressor [14], LUX (PCL1) act as an indirect activator of *CCA1/LHY*. The expression of *CCA1/LHY* is, therefore, constantly low in *lux/pcl1* mutants [10,11].

Since Prasinophyceae is an early-diverging class within the green plant lineage [16], the reduced circadian system in *O*. *tauri* [20,21] seems to have retained the primitive features of plant circadian clocks. The evening-loop may be the original position of GARP transcription factor in the plant circadian clocks, and the day-loop, such as ROC75, may be a newly acquired loop in the Chlorophyceae, including *C*. *reinhardtii*. Further comparative analyses among the three models, *C*. *reinhardtii*, *O*. *tauri* and *A*. *thaliana*, will provide insight into the evolution of the circadian clocks in the green plant lineage.

## Materials and methods

### Strains and transformation

In this study, we used the following *C*. *reinhardtii* strains: CBR (WT strain) [25], *roc75* and *roc114* (clock mutants [25]), ROC15-LUC, ROC40-LUC, ROC66-LUC, and ROC75-LUC (reporter strains expressing clock protein-luciferase fusion protein [35]). The CBR, *roc75*, and *roc114* strains have the *tufA-lucCP* reporter gene in their chloroplast genome [36]. The strains were maintained on agar plates of Tris-acetate-phosphate (TAP) medium [51] under constant illumination (10 μmol/m^2^/s).

The ROC75-HA, ROC75-YFP, ROC75-SRDX, ROC75-VP64 and ROC75-GR strains were generated by transformation of the *roc75* mutant. To generate transforming DNAs, artificially synthesized DNA fragments encoding HA, YFP, SRDX, VP64, and GR were cloned into the *Fse*I site of the pSI103PL/ROC75 plasmid [25] via ligation or In-Fusion assembly. The plasmids were digested with *Pac*I, and the fragments containing the tagged *ROC75* gene and the *aphVIII* selectable marker gene were used for transformation. The transformation was performed as described previously [35,52,53].

### Bioluminescence rhythm assay

The bioluminescence rhythm of spot cultures of reporter cells in white 96-well plates was monitored as described previously [25] except for the experiment in Fig 8B where we used spot cultures in black 24-well plates and a bioluminescence monitoring apparatus CL24-LIC (Churitsu Electric Corp., Nagoya, Japan). The circadian parameters (period, phase, and amplitude) were calculated by the cosinor method of the Rhythm Analyzing Program [54]. The amplitude was estimated from the overall average best-fitting cosinor curves between the second and the fifth day of bioluminescence monitoring. To monitor the clock protein-luciferase fusion reporters (Figs 2D, 6 and 7 and S3 Fig), we used AkaLumine-HCl (FUJIFILM Wako Pure Chemical Corporation, Osaka, Japan) [55] as the substrate because the bioluminescence rhythm was more stable against environmental disturbances (e.g., light on/off, drying of agar, vibration of culture plates).

### Genetic crosses

Genetic crosses were carried out as described previously [25]. The progeny were screened by bioluminescence and antibiotic resistance, and the genotype was confirmed by genomic PCR.

### Culture conditions

1) Batch TAP culture:

Cells were inoculated in TAP medium at a concentration of 1 × 10^5^ cells/mL and incubated at 24°C under LD conditions (12-h light:12-h dark; 30 μmol/m^2^/s) for 2–3 days.

2) Batch HS culture:

Two flasks of cells that were 12-h out-of-phase were prepared. The light and temperature schedules for LL, DD, and LD conditions are summarized in S11A–S11C Fig. The cells were inoculated into high-salt (HS) medium [56] at a concentration of 2 × 10^5^ cells/mL and incubated at 24°C under LL conditions (30 μmol/m^2^/s) for 3 days. After synchronization of the circadian clock employing both a temperature shift to 17°C and a 12-h dark exposure, the cultures were kept under the LL, DD, or LD condition. The cells were harvested at the indicated time points (24 to 72 h). The overlapping time points between the two flasks (36, 48, and 60 h) were used to validate the flask-to-flask variation. The sample collection during the dark period was done under safe, very low-intensity (less than 0.01 μmol/m^2^/s) blue LED light. Air was bubbled through the cultures exposed to the DD condition to ensure a stable circadian rhythm. No growth was observed even in the LL cultures (S11D Fig), ruling out the possibility of cell cycle-dependent rhythms.

3) Continuous culture:

The continuous culture was carried out as described previously [25].

### Western blot

Continuous culture (5 mL, 1-2x10^7^ cells) was harvested by centrifugation. Protein extraction and Western blot were carried out as described previously [39].

### Immunocytochemical staining

Batch TAP culture (1 mL, 0.5-1x10^7^ cells) was harvested by centrifugation. Immunocytochemical staining were carried out as described previously [39].

### RT-qPCR

Batch HS culture (5 mL, 1-2x10^7^ cells) was harvested by centrifugation. RNA extraction and RT-qPCR was carried out as described previously [39]. Data were normalized with respect to the expression level of *RCK1*. The sequences of primers used in the study are provided in S2 Table.

### YFP fluorescence imaging

A small aliquot (few microliters) of the batch TAP culture was dropped onto a cover slip and the drop was covered with a small piece (approximately 10 mm × 10 mm) of plastic wrap. *C*. *reinhardtii* cells were concentrated and immobilized in the wrinkles formed in the plastic wrap. The YFP fluorescence of the immobilized cells was observed with an inverted fluorescence microscope (IX70, Olympus) equipped with a CCD camera (DS-5M, Nikon).

### ChIP-seq and ChIP-qPCR

Cells (2.5x10^8^) were harvested from batch TAP culture by centrifugation. The cells were cross-linked for 5 min with 1% formaldehyde and then quenched by adding glycine to a concentration of 0.15 M. The cells were washed once with Tris-buffered saline and then lysed in ChIP lysis buffer (50 mM Tris-HCl pH 8.0, 10 mM EDTA, 1% SDS, 0.5 mM phenylmethylsulfonyl fluoride, 10 μL/mL of plant protease inhibitor cocktail [Sigma-Aldrich]). The chromatin was sheared by a sonicator (Bioruptor [CosmoBio, Tokyo, Japan]) at the maximum output for 30 sec for 30 cycles. The sonicated lysates were diluted ten times with ChIP dilution buffer (50 mM Tris-HCl pH 8.0, 167 mM NaCl, 1.1% Triton X-100, 0.11% sodium deoxycholate, 0.5 mM phenylmethylsulfonyl fluoride, 10 μL/mL of plant protease inhibitor cocktail [Sigma-Aldrich]). The HA-tagged protein-DNA complexes were precipitated with protein-G coupled magnetic beads (Dynabeads, Thermo Fisher Scientific) coated with an anti-HA antibody (MBL, Nagoya, Japan; 561–5, rabbit polyclonal). The precipitants were washed once with ChIP wash buffer I (50 mM Tris-HCl pH 8.0, 150 mM NaCl, 1 mM EDTA, 1% Triton X-100, 0.1% SDS, 0.1% sodium deoxycholate), once with ChIP wash buffer II (50 mM Tris-HCl pH 8.0, 500 mM NaCl, 1 mM EDTA, 1% Triton X-100, 0.1% SDS, 0.1% sodium deoxycholate), once with ChIP LiCl wash buffer (10 mM Tris-HCl pH 8.0, 0.25 M LiCl, 1 mM EDTA, 0.5% NP-40, 0.5% sodium deoxycholate), and twice with TE buffer (10 mM Tris-HCl pH 8.0, 1 mM EDTA), and then eluted with ChIP direct elution buffer (10 mM Tris-HCl pH 8.0, 300 mM NaCl, 5 mM EDTA, 0.5% SDS). After reversal of the cross-links by incubation at 65°C for 4 h, RNA digestion using RNaseA (20 μg/mL), and protein digestion using proteinase K (50 μg/mL), the DNA fragments were purified by phenol-chloroform extraction and ethanol precipitation.

The fragment libraries were constructed using the SOLiD fragment library construction kit (Thermo Fisher Scientific) according to the manufacture’s protocol, except for the purification steps that were carried out using the Agencourt AMPure XP Kit (Beckman Coulter). The pooled library was sequenced by multiplex paired-end sequencing on a flow cell using the 5500xl SOLiD system (Life Technologies, Waltham, MA, USA). The sequence reads were mapped to the reference genome of *C*. *reinhardtii* v 5.5 (Joint Genome Institute) [57] using Bioscope (version 1.2, Applied Biosystems SOLiD System). The total number of reads and mapped reads in each experiment are summarized in S3 Table. Mapping data has been registered in the DDBJ Sequence Read Archive (https://www.ddbj.nig.ac.jp/dra/index.html) under the accession numbers from DRR203090 to DRR203093. Peaks were detected by the peak calling algorithm for ChIP-seq analysis of CLC Genomics Workbench (version 7.5.1, Qiagen). To confirm the specificity of the detected peaks, we selected the peaks that were common in two biological replicates, and all the selected peaks were visually confirmed by comparing with the input DNA controls.

Quantitative PCR of ChIP DNA was performed using Fast SYBR Green I Master Mix (Thermo Fisher Scientific) and StepOnePlus (Thermo Fisher Scientific) according to the manufacture’s procedure. The primers used are provided in S2 Table.

### Preparation of the Glutathione-S-transferase (GST)-GARP protein and EMSA

The GST-GARP protein was expressed in the *E*. *coli* strain BL21 (DE3) cultured in LB and Terrific Broth media, supplemented with ampicillin (50 μg/mL). The cells were harvested by centrifugation, resuspended in 50 mM Tris-HCl buffer (pH 8.0) containing 50 mM NaCl, 1 mM EDTA, and 1 mM dithiothreitol (DTT), and disrupted by sonication. The lysates were clarified by centrifugation, and a GST-fused form of GARP was prepared from the lysates using Glutathione Sepharose 4B (GE Healthcare) and dialyzed for 12 h against 1 L of 20 mM Tris-HCl buffer (pH 8.0) containing 10 mM NaCl, 0.5 mM EDTA, and 1 mM DTT. The dialysates were applied to a Hitrap DEAE FF column (GE Healthcare) to purify the GST-GARP, and the proteins were further purified by ion-exchange chromatography on a MonoQ HR 5/5 column (GE Healthcare). The GST-GARP was then applied to a Hiload Superdex 75 10/300 GL column (GE Healthcare), equilibrated with 20 mM Tris-HCl (pH 7.5) containing 150 mM NaCl and 1 mM DTT. The concentration of proteins was determined using the Bio-Rad Protein Assay (Bio-Rad Laboratories); bovine serum albumin was used as a standard.

DNA and GST-GARP protein were incubated in Binding buffer (10 mM Tris pH 7.5, 0.1 mM EDTA, 100 mM NaCl, 0.1 mM dithiothreitol), and separated by non-denaturing polyacrylamide (5%) gel electrophoresis in 0.5X Tris-borate EDTA buffer. The DNA bands were stained with SYBR Green I (TaKaRa), and detected using Typhoon FLA9000 (GE Healthcare).

### *In vitro* luciferase assay

The cells were prepared in the same way as described for the bioluminescence rhythm assay. The cell spot on agar piece was flash frozen in liquid nitrogen in a 1.5-mL tube and stored at -80°C. The cell spots were lysed by adding 150 μL of Steady-Glo Luciferase Assay Reagent (Promega) and vortexed for 10 min. After the agar piece settled down, 80 μL of the supernatant was used for monitoring the luminescence in a multilabel plate reader (ARVO *X*4, PerkinElmer).

## Supporting information

S1 Fig Bioluminescence rhythms of the *roc114* mutant. **(A)** Bioluminescence rhythm of the WT and *roc114* mutant under the LD condition. Three representative bioluminescence traces of the *tufA-lucCP* reporter are shown. White and black bars above the graphs represent light and dark conditions, respectively. **(B)** The amplitude of rhythm. Bars represent means ± SD of 6–18 independent cultures. The asterisks indicate statistically significant differences (Student *t*-test; *P* < 0.001).
(EPS)

S2 Fig Generation of the ROC75-HA and ROC75-YFP strains. **(A)** A schematic view of the ROC75-HA and ROC75-YFP transgenes. White boxes represent exons. The positions of the 5′- and 3′-ends of the transcript are based on the latest genome annotation (*Chlamydomonas reinhardtii* v5.6, Phytozome 13, https://phytozome-next.jgi.doe.gov/). **(B)** Representative bioluminescence rhythms of the *tufA-lucCP* reporter. The number in the graph legend represents complemented-transformant/total-transformant-tested. White and black bars above the graphs represent light and dark conditions, respectively. **C**, Sequence of a codon-adapted YFP for the *C*. *reinhardtii* nuclear genome. A flexible GS-linker and restriction sites were attached.
(EPS)

S3 Fig Complementation of *roc75* arrhythmicity by the ROC75-LUC gene. The ROC75-LUC gene was introduced into the *roc75* strain by a genetic cross between ROC75-LUC (mt^+^) and *roc75* (mt^-^). White and black bars above the graphs represent light and dark conditions, respectively. Each point in the graph represents the mean ± SD of five biological replicates.
(EPS)

S4 Fig Location of peaks and the consensus motif of the ROC75-HA targets. **(A)** Pie chart of the peak location. **(B)** Consensus motif in the peaks. Consensus motif analysis of the detected peak regions was performed using MEME-ChIP (http://meme-suite.org/tools/meme-chip).
(EPS)

S5 Fig ChIP-qPCR analysis of ROC75-HA. Amplicons in the *ROC40* and *ROC55* (negative control) genes are shown on the top of the graph. The result of precipitation with normal IgG and precipitation from the WT strain are shown as negative controls. Bars in the graph represent means ± standard error of 3–4 independent experiments.
(EPS)

S6 Fig EMSA of the ROC75 GARP domain. **(A)** Schematic representation of the GST-tagged ROC75 GARP domain protein. **(B)** A representative result of EMSA. The sequence of DNA fragment used for the assay is shown on the top.
(EPS)

S7 Fig *In vitro* luciferase assay of the ROC15-LUC and ROC40-LUC strains. Cell spots of the reporter strains prepared, as well as those used in the *in vivo* bioluminescence rhythm assay, were harvested at the time points indicated and used for *in vitro* luciferase assay. The results of two biologically independent experiments are shown. The shaded areas in the main body of the graphs represent the time of day corresponding to the dark period for synchronization before the exposure to DD conditions.
(EPS)

S8 Fig Codon-adapted sequences of SRDX and VP64 for the *C. reinhardtii* nuclear genome. Sequences for SRDX (A) and VP64 (B) are shown. A triple FLAG tag, A flexible GS-linker, and restriction sites were attached.
(EPS)

S9 Fig A codon-adapted sequence of the hormone-binding domain of rat GR for the *C. reinhardtii* nuclear genome. The sequence corresponds to the amino acids from positions 508 to 794 of rat GR (GenBank, NP_036708). A flexible GS-linker and restriction sites were attached.
(EPS)

S10 Fig Representative traces of the bioluminescence rhythm of the *tufA-lucCP* reporter in ROC75-GR strain. DEX (final concentration: 2 μM) was added at the time-points indicated by arrows. The maximum of each trace was normalized to 100 for phase comparison.
(EPS)

S11 Fig Light and temperature schedules of batch HS culture for rhythm analysis. **(A-C)** Culture conditions for the LD (A), LL (B), DD (C) are shown. White and black bars represent light and dark conditions, respectively. Light intensities (μmol/m^2^/s) are indicated on the bars. **(D)** Cell number under the LL condition. Data points are means ± SD of at least three biologically independent experiments.
(EPS)

S1 Table List of detected genes by ChIP-seq analysis. (XLSX)

S2 Table List of primers. (XLSX)

S3 Table Number of sequence reads of ChIP-seq analysis. (XLSX)
